# Supplementary material for: High VEGFR3 Expression Reduces Doxorubicin Efficacy in Triple-Negative Breast Cancer
Source: Int J Mol Sci. 2023 Feb 10;24(4):3601. doi: 10.3390/ijms24043601 (PMC9966352; doi:10.3390/ijms24043601)
Supplement: Supplementary file 1 [file ijms-24-03601-s001.zip › ijms-2125822-supplementary.pptx]

## Slide 1
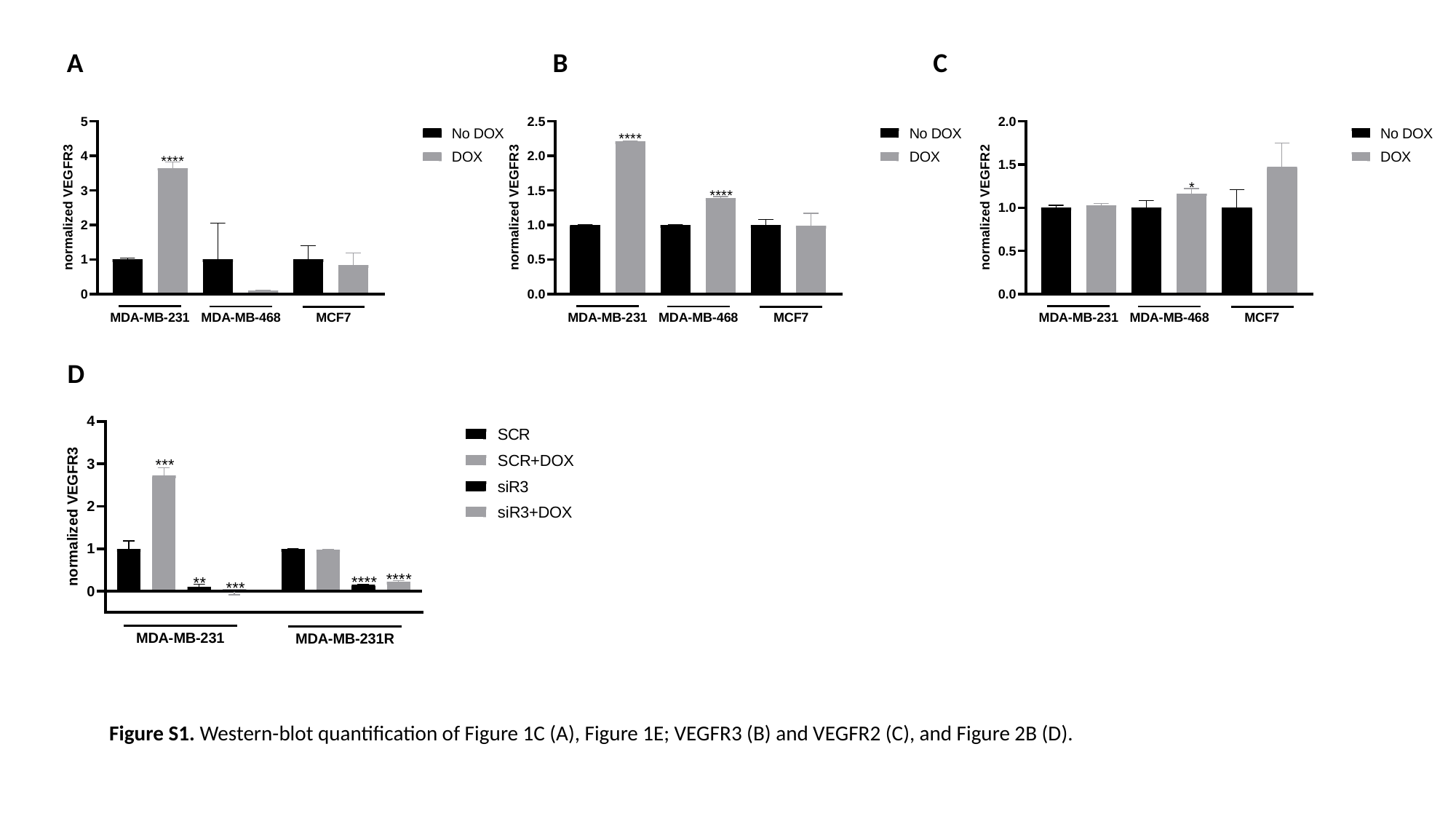

A
B
C
D
Figure S1. Western-blot quantification of Figure 1C (A), Figure 1E; VEGFR3 (B) and VEGFR2 (C), and Figure 2B (D).

## Slide 2
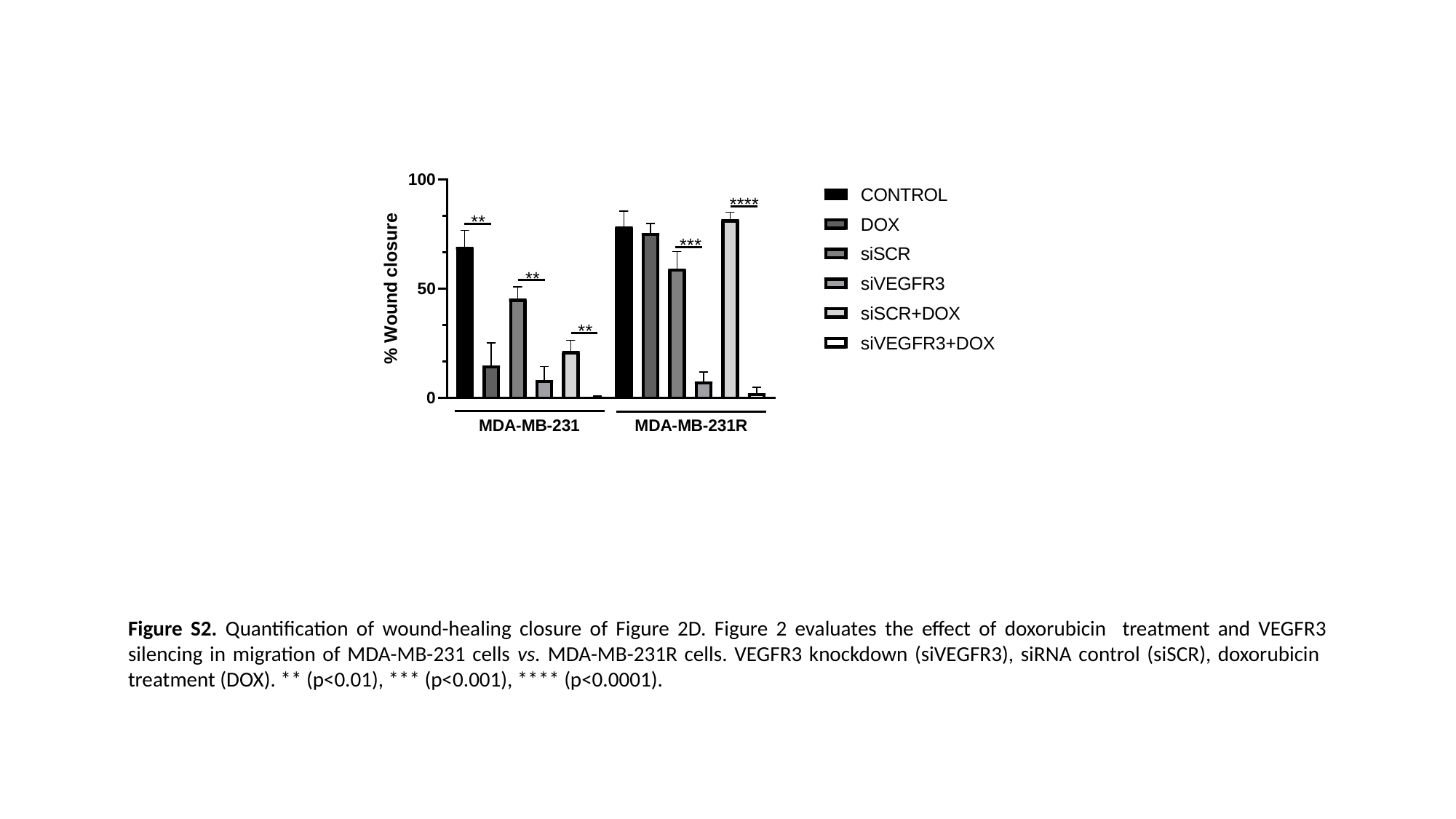

Figure S2. Quantification of wound-healing closure of Figure 2D. Figure 2 evaluates the effect of doxorubicin treatment and VEGFR3 silencing in migration of MDA-MB-231 cells vs. MDA-MB-231R cells. VEGFR3 knockdown (siVEGFR3), siRNA control (siSCR), doxorubicin treatment (DOX). ** (p<0.01), *** (p<0.001), **** (p<0.0001).
